# Neuromorphic device architectures with global connectivity through electrolyte gating

**DOI:** 10.1038/ncomms15448

**Published:** 2017-05-17

**Authors:** Paschalis Gkoupidenis, Dimitrios A. Koutsouras, George G. Malliaras

**Affiliations:** 1 Department of Bioelectronics, Ecole Nationale Supérieure des Mines, CMP-EMSE, MOC, Gardanne 13541, France

## Abstract

Information processing in the brain takes place in a network of neurons that are connected with each other by an immense number of synapses. At the same time, neurons are immersed in a common electrochemical environment, and global parameters such as concentrations of various hormones regulate the overall network function. This computational paradigm of global regulation, also known as homeoplasticity, has important implications in the overall behaviour of large neural ensembles and is barely addressed in neuromorphic device architectures. Here, we demonstrate the global control of an array of organic devices based on poly(3,4ethylenedioxythiophene):poly(styrene sulf) that are immersed in an electrolyte, a behaviour that resembles homeoplasticity phenomena of the neural environment. We use this effect to produce behaviour that is reminiscent of the coupling between local activity and global oscillations in the biological neural networks. We further show that the electrolyte establishes complex connections between individual devices, and leverage these connections to implement coincidence detection. These results demonstrate that electrolyte gating offers significant advantages for the realization of networks of neuromorphic devices of higher complexity and with minimal hardwired connectivity.

## Abstract (web summary)

Global regulation of synaptic strengths in neural systems is known as homeoplasticity. Here, Gkoupidenis *et al*. use an electrolyte to connect and control an array of organic electrochemical devices, in order to demonstrate behaviour that resembles homeoplasticity phenomena in the brain.

The main driving force for new developments in contemporary electronics is the evolution from increasing density to increasing functionality. It is now well recognized that electronic circuits based on traditional von Neumann architectures are not well-adapted to capture the real world information processing capability of biological nervous systems^1^. The main reason of this limitation is the so-called von Neumann bottleneck, due to the physical separation of computational and memory units^2^. Overcoming the von Neumann bottleneck can be achieved through the use of computational concepts borrowed from biology. Neuromorphic engineering, established in the late 1980s through the work of C. Mead^3^, involves mimicking the neuronal architectures present in the nervous system with silicon-based circuits. A recent breakthrough in the field is the development of neuro-inspired, multi-core chips with traditional CMOS (Complementary Metal/Oxide/Semiconductor) technology^4^. At the same time, a host of new devices with intrinsic neuromorphic behaviour gave birth to promising new computational paradigms that go beyond the traditional von Neumann architecture^5^. Several solid-state technologies including memristive, phase-change, spintronic and ferroelectric devices have been used in hardware-based implementations of the basic functional building blocks of neural processing and of the various forms of neuroplasticity^6^,^7^,^8^,^9^.

A recent trend in neuromorphic engineering involves the use of devices based on organic electronic materials^10^,^11^,^12^,^13^,^14^,^15^,^16^. This is largely motivated by the attractive characteristics organic devices offer in interfacing electronics with biology^17^. Indeed, the emerging field of organic bioelectronics is making available devices with exceptional performance for a variety of applications including neural interfaces, biosensors, and drug delivery, as well as new concepts such as chemical-based logic, and electronic plants^18^,^19^,^20^,^21^,^22^. A particularly popular example is the organic electrochemical transistor (OECT), a device that employs a channel made from an organic mixed electronic/ionic conductor, such as the conducting polymer poly(3,4ethylenedioxythiophene):poly(styrene sulf) (PEDOT:PSS). The electronic conductivity of the channel is modulated by ions injected from an electrolyte when an appropriate gate voltage is applied^23^,^24^. This mechanism of operation facilitates the strong coupling between electronic and ionic charge carriers and enables efficient communication between electronics and biological systems. It has also been used to demonstrate a variety of neuromorphic functions including short-, long-term synaptic plasticity functions and orientation selectivity^10^,^11^,^12^,^25^.

In the majority of neuromorphic circuits, each device is connected to others through a predefined physical wire network, a design that severely limits the number of connections that can be established between different devices^26^,^27^. On the other hand, biological information processing involves neural networks of immense connectivity^28^. The human brain for example consists of approximately 10^11^ neurons communicating with each other by an interwoven network of almost 10^14^ synapses, and each neuron might be connected to up to 10^4^ other neurons through these synapses. Synaptic weight expresses the connectivity degree between the neurons through the synapses and typically in computational neuroscience it can be defined as a scalar number between 0 and 1 (0—no connectivity from neuron to neuron, 1—maximum connectivity from neuron to neuron). The structure and the function of this complex network develops over time, resulting thus in memory and learning^28^. On top of all these, global mechanisms that modulate neural processing (that is, homeoplasticity) play a key role in brain function. Ionic concentrations, concentration of various hormones and temperature are some of the parameters that provide global regulation of synaptic strengths^29^,^30^. This homeostatic or global regulation of synaptic strengths is regarded as a normalization mechanism that is necessary for the stabilization of correlation-based rules such as Hebbian plasticity^29^,^31^. As positive feedback rules tend to destabilize the behaviour of large neural networks, homeostatic phenomena force globally specific constraints in synaptic strengths^31^,^32^,^33^,^34^.

In this work, we demonstrate the concept of global regulation in neuromorphic device architectures through an electrolyte, a behaviour analogous to homeostasis in the brain (Fig. 1a). We use an array of two-terminal devices immersed in an electrolyte, where each device consists of a PEDOT:PSS channel that serves as a hard connection between an input and an output wire. We show that the weights of these hard connections are modulated globally by the voltage applied on the electrolyte and by its ion concentration, in the way that is analogous to homeoplasticity. We take advantage of this effect to demonstrate synchronization of I/O transmission and global clock behaviour, reminiscent of the coupling between individual neuron activity and global oscillations in biological networks. We further show that the electrolyte establishes complex soft connections (that is, without physical wiring) between individual devices. We leverage these connections to implement coincidence detection, a function used in the visual and auditory systems of the brain, in devices with minimal hard connectivity. These results demonstrate that electrolyte connections offer significant advantages for the realization of networks of neuromorphic devices of higher complexity.

## Results

### The concept of global gating

**Figure 1:**
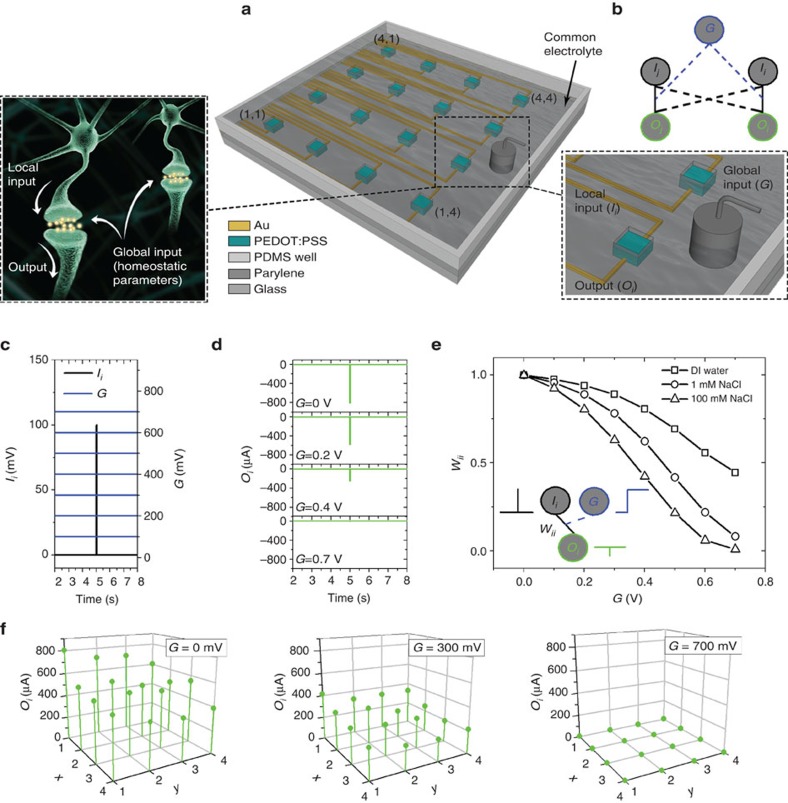
Configuration of the array and principle of global input. (**a**) Schematic of the configuration of the array of PEDOT:PSS devices. Each device consists of a PEDOT:PSS channel connected with two Au wires, the local input *I*_*i*_ and the output *O*_*i*_. The PEDOT:PSS channels are in contact with a common electrolyte in which a Ag/AgCl electrode serves as the global input *G*. The analogy of the device grid and a system of neurons that are immersed in a common electrolyte is also shown. In neural environment global (homeostatic) parameters regulate the overall function of the brain. (**b**) Representation of the physical (straight line) and soft connections (dash line) that are established in an array of two devices that are immersed in a common electrolyte. (**c**) Combinations of local and global inputs (*I*_*i*_: pulse of amplitude of 100 mV and width of 50 ms, *G*: DC voltage 0–700 mV), and (**d**) resulting outputs. (**e**) Connection weight *w*_*ii*_ as a function of the global gate voltage *G* for different electrolyte concentrations (DI water—100 mM NaCl). Inset shows that weight *w*_*ii*_quantifies the physical connectivity between input *I*_*i*_ and output *O*_*i*_, and that it is modulated by the global input. (**f**) Spatial maps showing the variation of outputs *O*_*i*_, when voltages pulses (amplitude of 100 mV, width of 50 ms) were used at each local input *I*_*i*_, and various DC values (0, 300 and 700 mV) were used as a global input. The Ag/AgCl electrode was located as shown in (**b**). The maps show that global input forces a global restriction on every output.

The device architecture is depicted in Fig. 1a to consist of a 4 × 4 grid of two-terminal devices in a square configuration, where indexes *i*=1–4 and *j*=1–4 correspond to device number on the grid. As will be presented below, various physical and soft connections can be established between the terminals of the devices and through the electrolyte (Fig. 1b). The fabrication process is described in the experimental section. Briefly, each device consists of a lithographically defined PEDOT:PSS channel that connects two Au wires deposited on a glass substrate. The channels have nominal dimensions of 50 μm × 50 μm (length × width, *L* × *W*) and are placed at a centre-to-centre device distance *d*=5 mm. The Au wires are covered by an insulating film, therefore only the PEDOT:PSS channels are exposed to a common 100 mM (unless otherwise stated) NaCl electrolyte in which a Ag/AgCl gate electrode is immersed. For each device, one of the Au wires is used as a local input (*I*_*i*_) and a voltage is applied as the input signal. The other terminal is connected to the ground through an electrometer, and the measured current is defined as the output (*O*_*i*_). Each device can also be addressed by the global input (*G*) when a voltage is applied to the gate electrode.

Figure 1c–f show how the global input can be used to modulate individual devices. The protocol for the measurements is displayed in Fig. 1c and consists of applying voltage pulses at the local input *I*_*i*_, while applying various DC voltages at the global input *G*. The output *O*_*i*_ of the device at coordinates (1, 1) is shown in Fig. 1d to be suppressed as the global input increases. This can be described as in Fig. 1e, by considering that the connection weight *w*_*ii*_ (defined as *O*_*i*_/*O*_MAX,*i*_, where *O*_MAX,*i*_ is the output that corresponds to a global input of 0 V) is gradually suppressed by the action of the global input and becomes practically zero for large positive values and for an electrolyte concentration of 100 mM. It should be mentioned that the term connection weight is used here instead of the classic definition of synaptic weight, because it has volatile nature and is a metric of how the physical connection can be modulated by using a global gate electrode. The effect of the global input is also seen in Fig. 1f, which shows the outputs *O*_*i*_ of all devices on the array to be massively suppressed as global gate voltage increases. It should be noted that the variation of *O*_*i*_ (Fig. 1f) is attributed to the resistance of the metal connections, which are longer for the devices (*i*,1) than (*i*,4). Moreover, the influence of the global gate is modulated by the ion concentration in the electrolyte. As seen in Fig. 1e, when the electrolyte concentration is decreased, the influence of the global gate is becoming weaker. Indeed, the output *O*_*i*_ is only partially suppressed by the global gate when DI water is used. Detailed gate voltage versus electrolyte concentration spatial mappings of the local output *O*_*i*_, presented in Supplementary Fig. 1 (refer also to Supplementary Note 1), show this to happen in all devices of the array. Both gate electrode and electrolyte concentration force global restrictions to the output of every device, emulating in this way homeoplasticity phenomena. This global restriction behaviour is practically independent on the exact location of the gate (see also Supplementary Fig. 2 and Supplementary Note 2).

These effects can be understood using the standard model for OECTs, according to which cations are injected from the electrolyte to the channel under the combined influence of the voltage applied at the global and local inputs and decrease the conductivity of the PEDOT:PSS film. This phenomenon can be modelled using an equivalent circuit^24^,^35^. In the simplest form, this circuit consists of a resistor, representing ionic transport in the electrolyte, in series with a capacitor, representing the charging of PEDOT:PSS with ions. The resistor increases with decreasing ion concentration in the electrolyte, and this increases the RC time and decreases the efficiency of the global gate. Moreover, the influence of various electrolytes on the operation of PEDOT:PSS based devices has also been studied at past^36^,^37^.

### Synchronization functions

**Figure 2:**
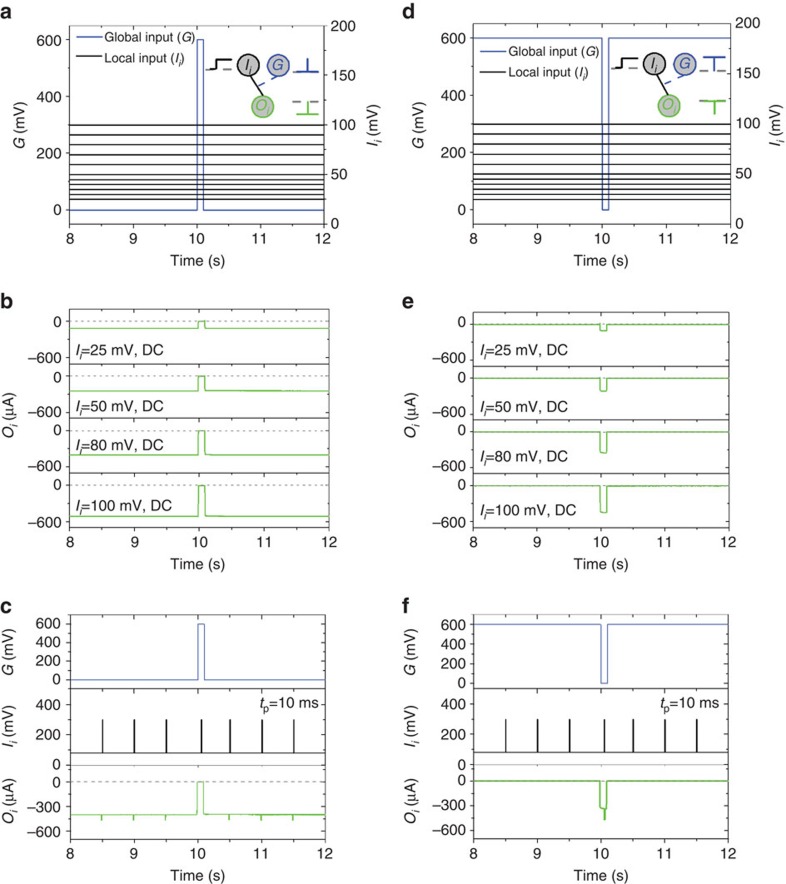
Synchronization of I/O transmission with a global input. (**a**) Combinations of local and global inputs (*I*_*i*_: DC voltage 25–100 mV, *G*: pulse of amplitude of 600 mV and width of 50 ms), and (**b**) resulting outputs, showing that *I*_*i*_/*O*_*i*_ transmission shuts down when a voltage pulse is applied at the global input. (**c**) The same pulse shuts down transmission of voltage pulses (amplitude of 220 mV, offset of 80 mV, width of 10 ms) applied at the local input. (**d**) Combinations of local and global inputs, now with an inverted pulse as the global input, and (**e**) resulting outputs showing that transmission is enabled during the inverted pulse. (**f**) The same pulse now enables transmission of local input.

**Figure 3:**
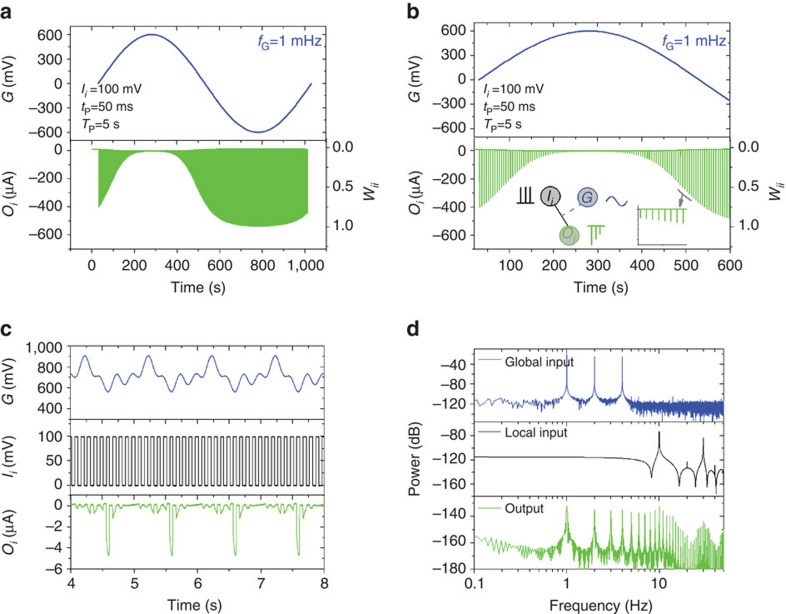
Global input as a global clock. (**a**,**b**) Periodic modulation of the output caused by a train of voltage pulses (amplitude of 100 mV, width of 50 ms, period of 5 s) at a local input, and a sine wave (amplitude of ±600 mV, frequency of 1 mHz) at the global input, shown here for a full period (**a**) and for the positive half of the cycle (**b**). (**c**) Global input with higher harmonics (amplitude modulation of a sine wave of frequency of 1 Hz, amplitude of 180 mV and offset of 700 mV, with a second sine wave of frequency of 3 Hz, AM depth of 120%), and a train of voltage pulses (amplitude of 100 mV, width of 50 ms, period of 100 ms) at local input results to an output that contains predominantly the low-frequency components of the global input. (**d**) Power spectra of *G*, *I*_i_ and *O*_*i*_.

The global input can be used to impose synchronization of I/O transmission. As shown in (Fig. 2a,b), a voltage pulse at the global input temporarily shuts down transmission of the local input of the device at coordinates (1, 1). This behaviour can be utilized to synchronize globally the I/O transmission of an array of devices. For example, in Fig. 2c, a train of voltage pulses at the local input is transmitted to the output, unless a voltage pulse is applied at the global input at the same time (which happens at *t*∼10 s). Figure 2d–f shows that an inverted pulse at the global input has the opposite effect, namely enabling local I/O transmission during its presence. In Fig. 2c,f, a train of pulses is used as local input with amplitude of 220 mV, offset of 80 mV, width of 10 ms, in order to study the I/O transmission. The same phenomenon can be used to implement a global clock. As shown in Fig. 3a,b an oscillatory voltage at the global input modulates a train of voltage pulses at the local input. Indeed, the connection weight *w*_*ii*_ is modulated within the entire range (from 0 to 1), and in a purely analogue manner. This happens simultaneously for all the devices in the array (Supplementary Fig. 3, Supplementary Note 3), hence the oscillation acts as a global clock, allowing transmission of local input signals only during certain periods of time.

Synchronization is also achieved when a voltage oscillation containing higher harmonics is used as the global input. In Fig. 3c the global input imposes periods of time during which I/O transmission suppressed, and this permits the synchronization of a local input signal with a global oscillation at its basic harmonic of 1 Hz. More specifically, a global oscillation chosen with a positive offset is chosen (in the range 500–900 mV), in order to operate the device in the sub-threshold regime (that is, *G*>500 mV, or the ‘OFF' state) during the majority of the global oscillation, and only during the basic harmonic of the AC signal (that is, 1 Hz) the device is operated close to the threshold regime (*G*∼500 mV). This permits the implementation of synchronization functions. Power spectra of the input and output signals are shown in Fig. 3d. The spectral density of the output signal displays the basic harmonics of the global oscillation, which are the 1, 2 and 4 Hz (see also Supplementary Note 4). The spectral density also shows a range of higher frequencies along with the basic harmonics, due to the need of a whole range of harmonics for the construction of the complex structure of the current output *O*_*i*_ of Fig. 3c. Moreover, when a steeper oscillation is used as a global signal, this results in higher output current *O*_*i*_ (see also Supplementary Fig. 4). The low-frequency harmonics of the global input are transmitted to the output. Therefore the global input plays the role of a synchronization function similar to that in biological systems, where low-frequency global neural oscillations provide discrete activation windows for the neural response^38^,^39^. Notably, the behaviour of Fig. 3c,d also resembles the phenomenon of neural entrainment, which is related to the synchronization of the activity of neural ensembles with periodic external stimuli (that is, acoustic or optical)^40^,^41^.

### Soft connections thought the electrolyte

**Figure 4:**
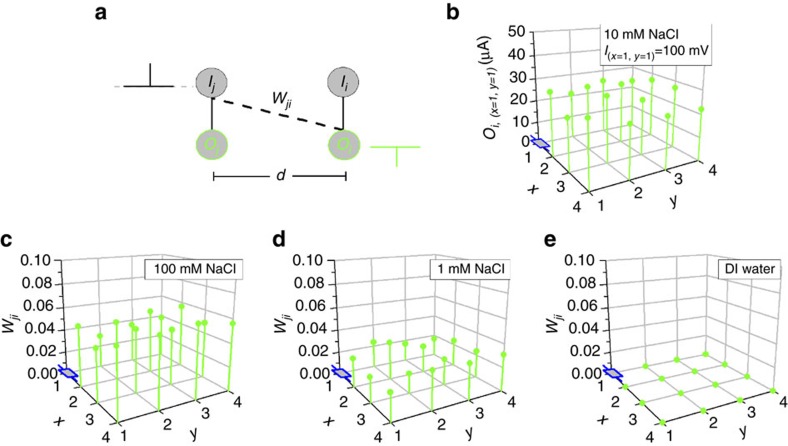
Lateral communication of devices through soft electrolyte connections. (**a**) Schematic of lateral communication and definition of the soft connection weight *w*_*ji*_. (**b**) Spatial map of the output *O*_*i,* (*x=*1, *y=*1)_ of the device at coordinates (1, 1) when a pulse is applied at local input *I*_*j (x*≠1*, y*≠1)_ (amplitude of 100 mV, width of 50 ms) of devices at coordinates *x*≠1, *y*≠1. The distance *d* between the devices is 5 mm. (**c**–**e**) Spatial mapping of the connection weight *w*_*ji*_ (defined as *O*_*i*_/*O*_MAX, *i*_) for different electrolyte concentrations (100 mM, 1 mM, DI water).

In addition to providing a global input, the electrolyte also introduces soft connections between the inputs and outputs of different devices and enables their communication. As described in Supplementary Note 5, effectively each device acts as a gate for all the other devices (see also Supplementary Fig. 5). The concept is demonstrated in Fig. 4a, where the input *I*_*j*_ of the *j*th device also acts as an input to an adjacent *i*th device, with the coupling between the *I*_*j*_ input and the *O*_*i*_ output characterized by the connection weight *w*_*ji*_. Here the term connection weight is used to express the correlation of a corresponding lateral output in respect to a physical output. A spatial map of the output *O*_*i*_ of the device at coordinates (1, 1), that results from applying an input pulse at every local input *I*_*j*_, of devices of the array is depicted at Fig. 4b. The results show that a local input has an effect on all devices of the array. The spatial mapping of the connection weight *w*_*ji*_ (defined here as *O*_*i*_/*O*_MAX,*i*_, where *O*_MAX,*i*_ is the output that results from each corresponding *i*th local input *I*_*i*_) is depicted in Fig. 4c. When compared to the physical connection weight *w*_*ii*_ (see also Fig. 1d), the soft connection weight *w*_*ji*_ is weaker. For example, a soft connection between a *j*th local input and an *i*th output, creates an output *O*_*i*_ that is ∼5% of the output elicited by the corresponding local input. Figure 4c–e show the spatial mappings of *w*_*ji*_. Similarly to hard connection weights, the soft connection weight *w*_*ji*_ also depends on the electrolyte concentration. As the electrolyte concentration decreases, the weight *w*_*ji*_ decreases (from ∼5% *O*_MAX,*i*_ for 100 mM to ∼2% *O*_MAX,*i*_ for 1 mM), and in the case of DI water, lateral connectivity between individual devices is completely suppressed (w_ji_∼0% *O*_MAX,*i*_).

### Coincidence detection

**Figure 5:**
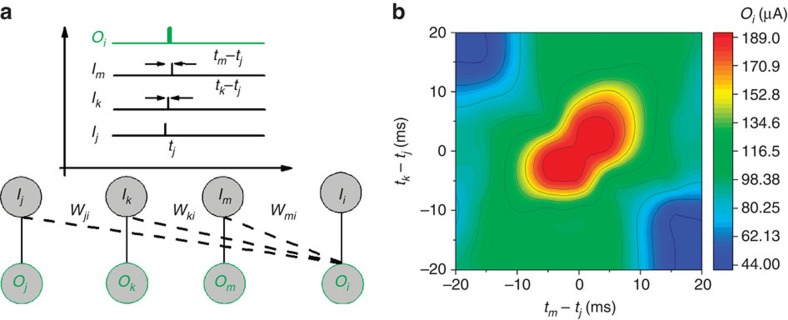
Coincidence detection using soft connections between devices. (**a**) Schematic of the measurement protocol. A voltage pulse (amplitude of 0.5 V, width of 10 ms) is applied at three local inputs *I*_*j*_, *I*_*k*_, *I*_*m*_ with variable time intervals *t*_*k*_−*t*_*j*_ and *t*_*m*_−*t*_*j*_ with respect to a reference time *t*_*j*_ of the input *I*_*j*_. The output *O*_*i*_ of the *i*th device is measured for different timing conditions. (**b**) Time correlation map of the output *O*_*i*_ showing coincidence detection.

The availability of soft lateral connections between devices endowed by the electrolyte can be leveraged to realize multi-terminal functionalities from individual devices without complex wiring. This behaviour has important implications in neuromorphic computing because in this way, complex summations from multiple inputs can be performed in another output through the electrolyte continuum without predefined physical connections. In biological neural networks the parallelism originates from the massive interconnections between the neurons through the synapses. The concept of interconnectivity is very important in the actual neural environment and is a key feature for reliable computing with unreliable neurons, in which there is a redundancy of interconnections. Figure 5a,b demonstrates the way that time correlated signals from various spatially distributed local inputs are superimposed in a separate output (*O*_*i*_). Here, voltage pulses are applied at three local inputs (*I*_*j*_, *I*_*k*_, *I*_*m*_) with variable time intervals *t*_*k*_−*t*_*j*_ (for the *I*_*k*_ input) and *t*_*m*_−*t*_*j*_ (for the *I*_*m*_ input) with respect to a reference time *t*_*j*_ of the *I*_*j*_ input (Fig. 5a), and the output *O*_*i*_ of the *i*th device is measured (refer also to Supplementary Fig. 6 and Supplementary Note 6). Following the above mentioned measurement protocol, a time correlation map of the output *O*_*i*_ is constructed and depicted in Fig. 5b. The map shows that a maximum output *O*_*i*_ results when all inputs *I*_*j*_, *I*_*k*_, *I*_*m*_, are time synchronized (that is, when *t*_*m*_−*t*_*j*_, *t*_*k*_−*t*_*j*_∼0). Secondary maximums of the *O*_*i*_ also appear on the map, which are attributed to the synchronization of just two inputs. The overall output response *O*_i_ is slightly super-linear summation of the individual local inputs (see also Supplementary Fig. 7 and Supplementary Note 7). The occurrence of spatially distributed, multiple inputs is encoded through soft connections in the response of *O*_*i*_, a behaviour that resembles coincidence detection in biological neural networks. Coincidence detection neurons play important role in neural signalling, especially in the visual^42^, and auditory system^43^,^44^. In the binaural cochlear for example, coincidence detection is regarded as a mechanism for conversion of acoustic to spatial codes and it is thus used in the auditory system for spatial localization. The fact that such multi-terminal functionalities are implemented by taking advantage of soft connections in a device array with minimal hard wiring highlights the utility of electrolyte as connectivity media in neuromorphic systems.

## Discussion

Previous papers on neuromorphic devices explored the role of electrolytes in connecting one^10^,^11^, or multiple inputs to a single device^12^,^45^,^46^. In this work, we use a common electrolyte to connect and control a whole array of devices. The PEDOT:PSS channels represent hardwired connections between inputs and outputs, and their connection strengths can be globally modulated by the applied voltage and by the ion concentration in the electrolyte. This enables slowly-varying AC voltage signals at the electrolyte to be used as global clocks that regulate periodically the I/O transmission. Other global clocks, such as periodic changes in ionic concentration, are also expected to regulate the I/O transmission and connection weights and this might lead to applications in biosensing. Most importantly, electrolyte gating is shown to exhibit global inhibitory characteristics and is used to massively suppress connectivity in the entire array. This is a key feature for neuromorphic devices, in order to emulate homeostatic plasticity phenomena of the neural environment^29^,^31^.

In this work, we use volatile PEDOT:PSS devices in order to provide the simplest demonstration of global gating. Other PEDOT-based derivatives that exhibit memory phenomena^11^,^47^, can be used to introduce long-term-memory phenomena and lead to more complex network behaviours. Moreover, the performance of the architecture discussed here can be tuned through the use of anisotropic ionic conductors^48^ as global electrolytes, which are expected to define spatial- or directional-dependent soft connection weights. Furthermore, ion-selective conjugated polymers as active materials^49^, can be leveraged to implement unique features such as local processing of ionic signals from various ionic species. Finally, the phenomena described here, such as the influence of applied voltage and ion concentration in the electrolyte on conductivity of PEDOT:PSS, have been studied in conjunction with the development of OECTs, and quantitative models exist to describe them. The global gate/electrolyte/PEDOT:PSS stack, for example, is identical to the ionic part of the circuit of an OECT, which is modelled using simple equivalent circuits. The availability of such models allows the fine tuning of the response of neuromorphic devices in order to for example, access specific timescales required in particular applications.

## Methods

### Device fabrication

The devices were fabricated using standard microfabrication techniques. As substrates, 26 × 76 mm glass slides were used. The contact lines were defined by evaporating a 10 nm Cr and a 100 nm Au layer on top of pre-patterned photoresist and a subsequent soaking in an acetone/isopropanol bath to remove the excess material. The Cr layer is needed to enhance surface adhesion between glass and Au. To protect the contact lines from the electrolyte, two parylene C layers were deposited on top, with 2 μm thickness each. The first was deposited on a surface treated with silane to enhance adhesion with the substrate and a thin 2% soap solution layer was spin-coated before the second deposition. This allows to peel-off the upper layer, thereby defining the device active area with nominal dimensions of 50 μm × 50 μm (length × width, *L* × *W*). The polymer used in this communication was PEDOT:PSS [Clevios PH 1,000 from Heraeus Holding GmbH, with 5 wt% ethylene glycol, 0.1 wt% dodecyl benzene sulf acid and 1 wt% of (3-glycidyloxypropyl) trimethoxysilane)]. Three subsequent spin-coating steps with varying rotation speeds of 1,500, 650 and 650 r.p.m., respectively, yielded a film thickness of ∼500 nm. A second photolithographic step was used to pattern these features. PEDOT:PSS spin-coating was followed by a hard**-**bake step at 140 °C for 60 min. Using the above mentioned process, an array of 4 × 4 devices was fabricated with centre-to-centre distance *d*=5 mm. For every input and output terminal, metal pads that extend outside the electrolyte region and were not covered with parylene were used.

### Device characterization

The array of 4 × 4 devices was gated with a global Ag/Cl gate electrode and aqueous NaCl electrolyte (1–100 mM) in deionized water. Measurements were recorded after cycling the every device (repetitive 0.3/−0.3 V cycles at the gate for 2 s each) in order to obtain reproducible behaviour (endurance measurements are presented in Supplementary Fig. 8 and Supplementary Note 8). The current of the *O* terminal was measured using a National Instrument PXIe-1062Q system. The devices were biased with a PXIe-4145 source measure unit that was simultaneously recording the current at the output with a sampling rate of 1 kHz. Voltages for the local and global inputs were generated by a National Instruments USB-6259. Both *G*, *I* voltages and *O* current measurements were internally triggered by the PXIe system. For the measurement of soft connectivity, a bias of −50 mV was applied to the device whose output was measured, and the global gate electrode was disconnected. The input signal containing higher harmonics was generated with a Keithley 3,390 Arbitrary Function Generator. The acquisition system was controlled by custom-made LabVIEW software. Fast Fourier Transform was performed using OriginPro software.

### Data availability

The data that support the findings of this study are available from the corresponding authors upon request.

## Additional information

**How to cite this article:** Gkoupidenis, P. *et al*. Neuromorphic device architectures with global connectivity through electrolyte gating. *Nat. Commun.* **8,** 15448 doi: 10.1038/ncomms15448 (2017).

**Publisher's note**: Springer Nature remains neutral with regard to jurisdictional claims in published maps and institutional affiliations.

## Supplementary Material

Supplementary Information Supplementary Figures, Supplementary Notes and Supplementary References
