# Supplementary material for: Neuromorphic device architectures with global connectivity through electrolyte gating
Source: Nat Commun. 2017 May 17;8:15448. doi: 10.1038/ncomms15448 (PMC5442355; doi:10.1038/ncomms15448)
Supplement: Supplementary Information — Supplementary Figures, Supplementary Notes and Supplementary References [file ncomms15448-s1.pdf]

Supplementary Figures

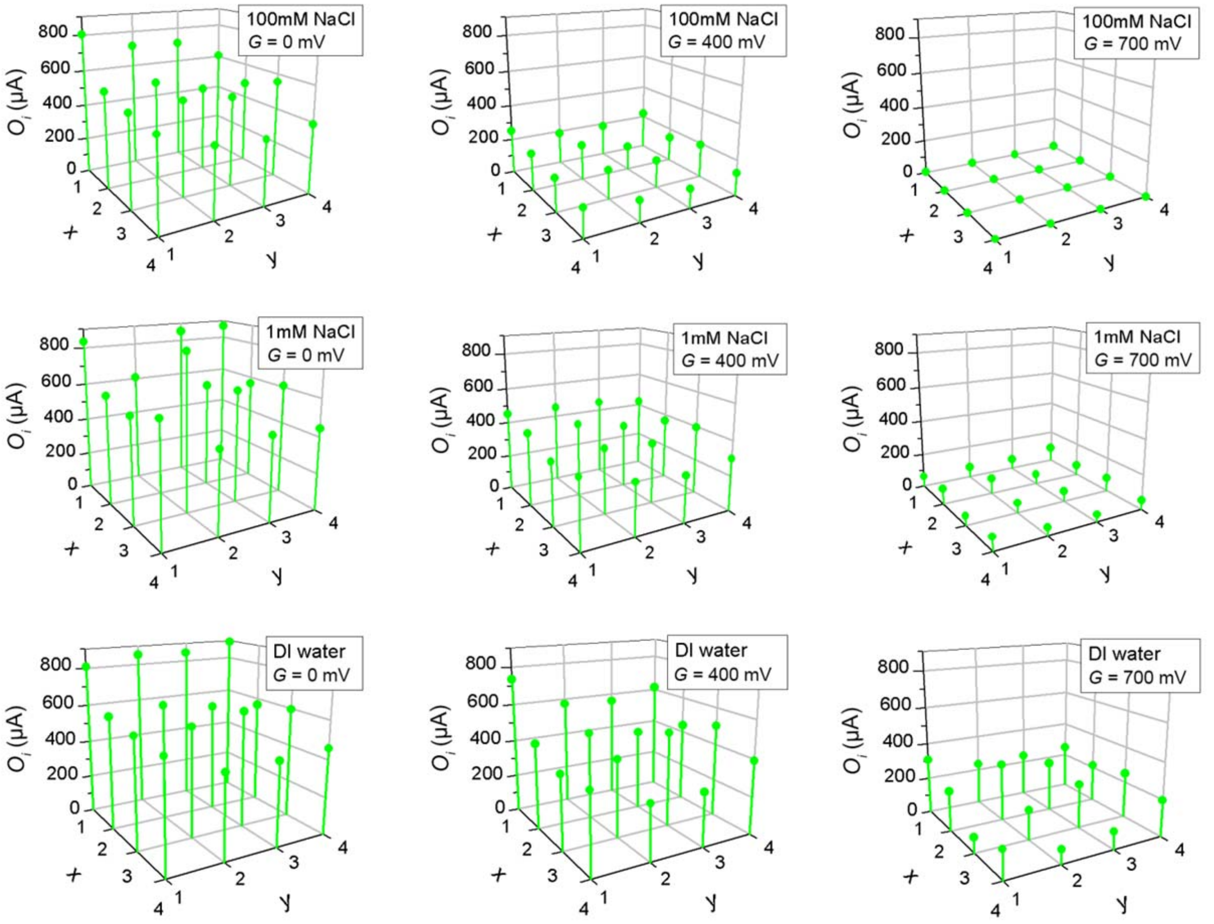

Supplementary Figure 1. Gate voltage vs electrolyte concentration spatial mappings of output O_i. A pulse is applied at every local input I_i (amplitude of 100 mV, width of 50 ms) and the amplitude of the corresponding output O_i is determined for different global DC gate voltages (G = 0 – 700 mV) and electrolyte concentrations (DI water – 100 mM NaCl). More effective global gating is observed through the 'diagonal' direction of the mappings (for high voltages and high electrolyte concentrations).

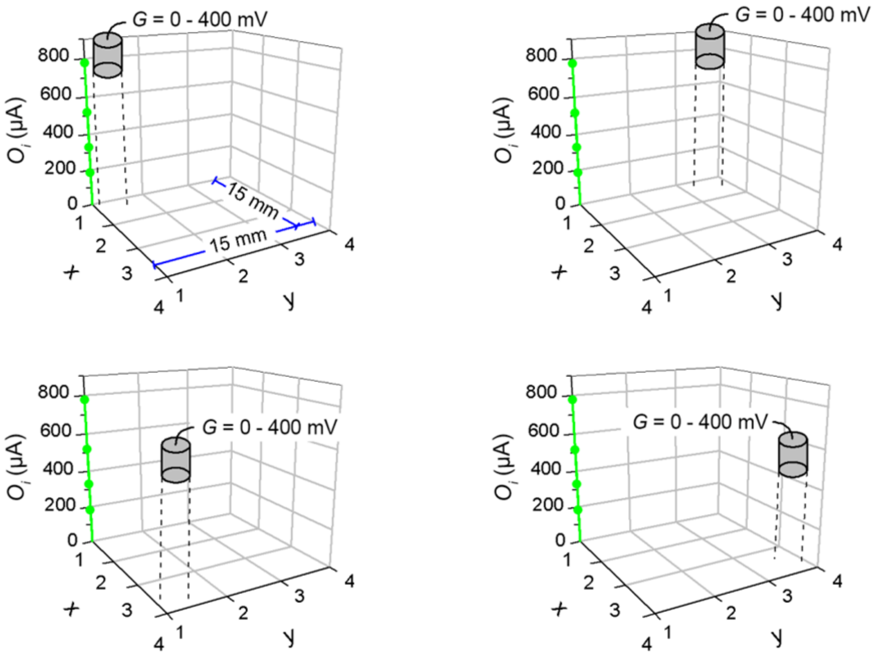

Supplementary Figure 2. Influence of the gate-device distance on global gating. Output O_i of the device at coordinates (1, 1) for the application of a pulse at the local input I_i (amplitude of 100 mV, width of 50 ms), global gate voltages $G = 0 – 400$ mV and various gate-channel distances on the 4 × 4 grid.

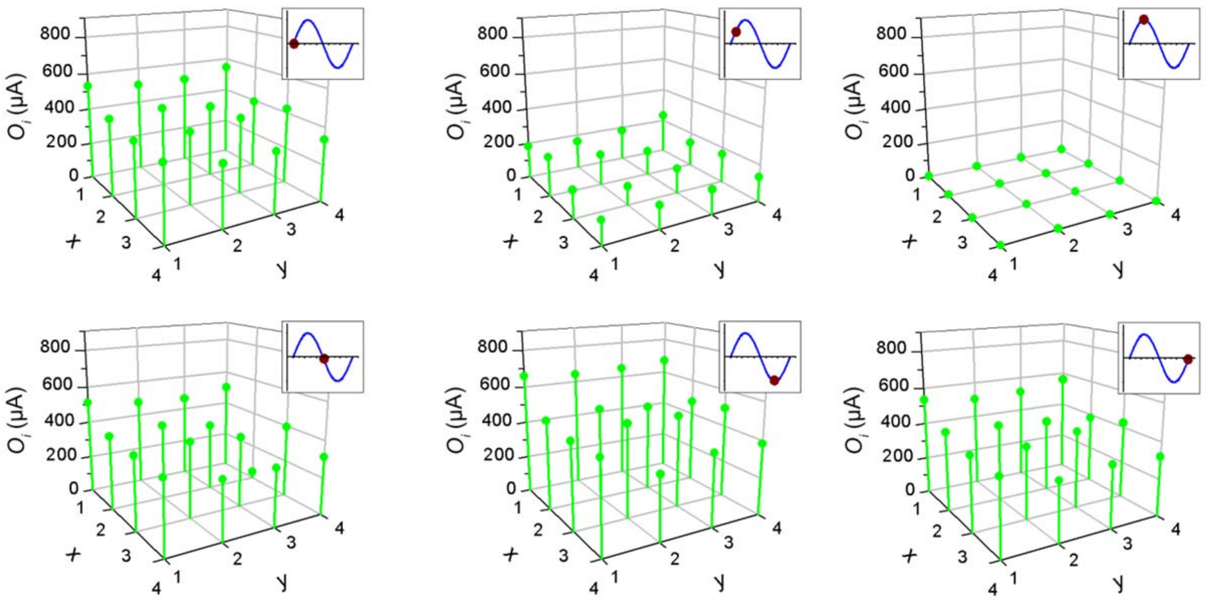

Supplementary Figure 3. Gating the device grid in a global electrolyte with an AC signal. Spatial output current mapping of O_i, at different moments of time of the AC global signal (frequency of 1 mHz and amplitude 600 mV). A voltage pulse is applied at every local input I_i (amplitude of 100 mV, width of 50 ms) and the output current O_i of every device is measured as a function of time. Every graph corresponds to a moment of time of the AC signal (inset).

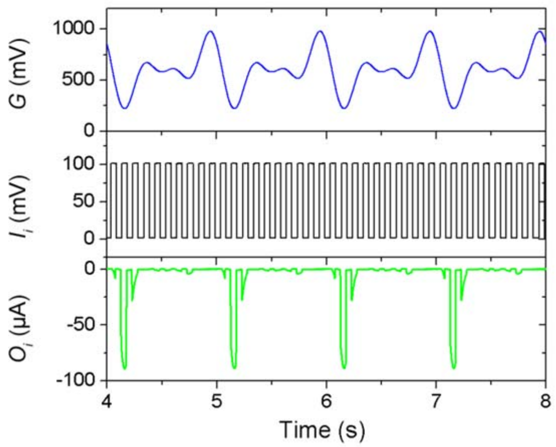

Supplementary Figure 4. Synchronization function. Resulting output current O_i, with the application of a global input G with higher harmonics (amplitude modulation of a sine wave of frequency of 2 Hz, amplitude of 500 mV and offset of 600 mV, with a second sine wave of frequency of 1 Hz, AM depth of 120%), and a train of voltage pulses (amplitude of 100 mV, width of 50 ms, period of 100 ms) at local input I_i. The steeper oscillation leads to higher output current O_i.

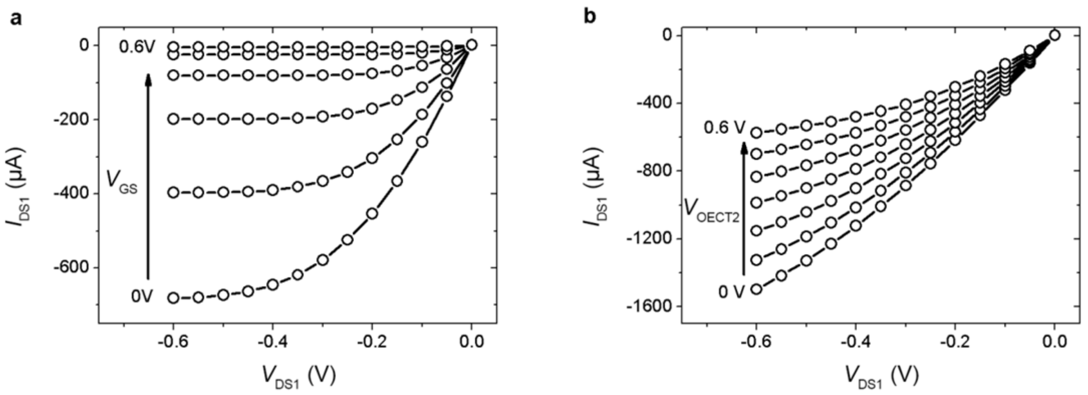

Supplementary Figure 5. OECT characteristic curves. (**a**) I_{DS} – V_{DS} measurements in typical 3-terminal transistor configuration for the OECT1 using a AgCl gate electrode (V_{DS} = 0 - -0.7 V, V_{GS} = 0 – 0.6 V). (**b**) I_{DS} – V_{DS} measurements in typical 3-terminal transistor configuration for the OECT1 using another transistor OECT2 as a gate electrode (V_{DS} = 0 - -0.7 V, V_{OECT2} = 0 – 0.6 V).

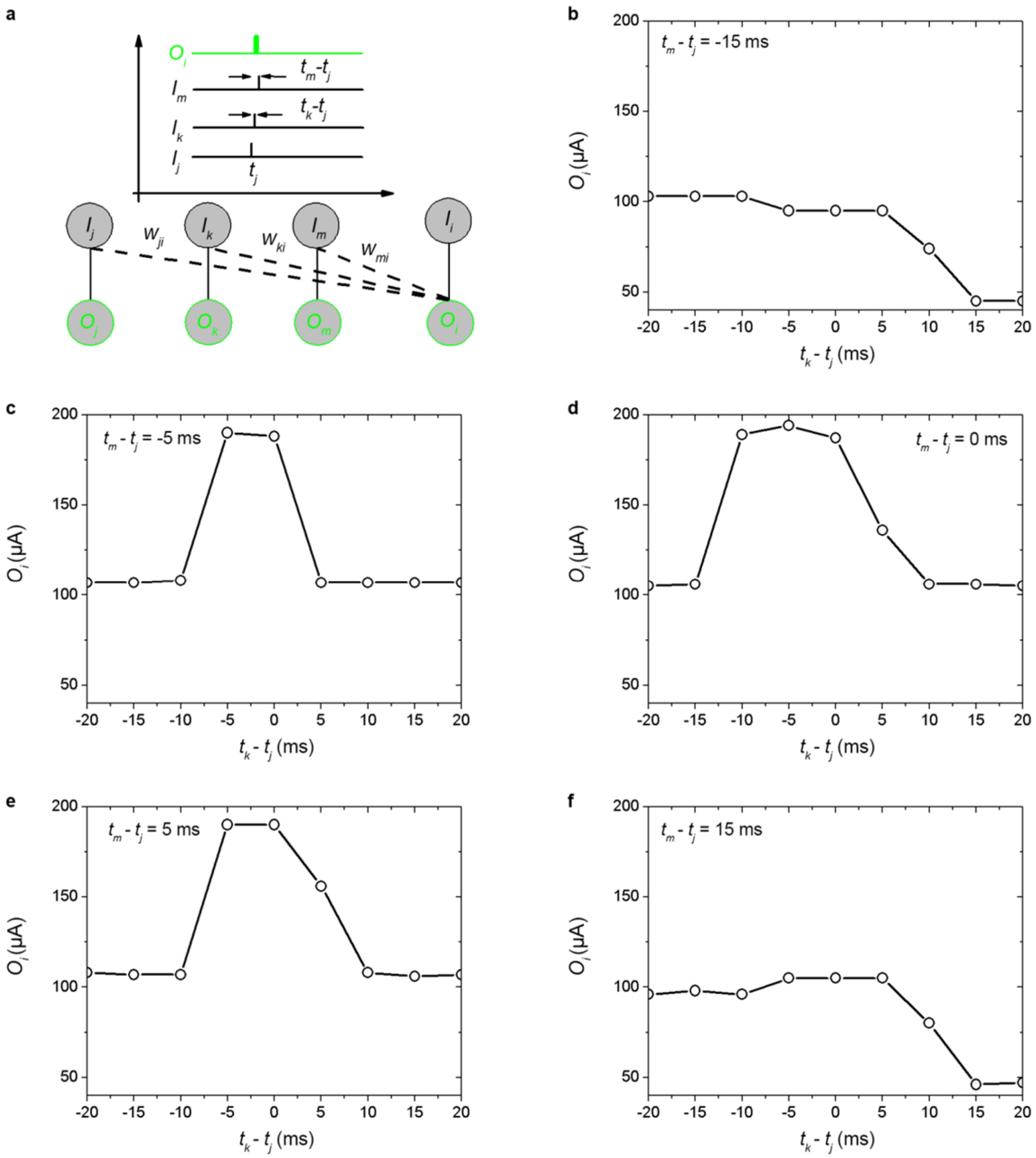

Supplementary Figure 6. Time correlation mapping. (**a**) Schematic of the measurement procedure. (**b**)-(**f**) Amplitude of O_i vs $t_k - t_j$ for different $t_m - t_j$ time intervals ($t_m - t_j$ = -15 ─ 15 ms). Time correlation mapping of Fig. 5(b) is constructed by combining the graphs of Supplementary Figure 3(b)-(f).

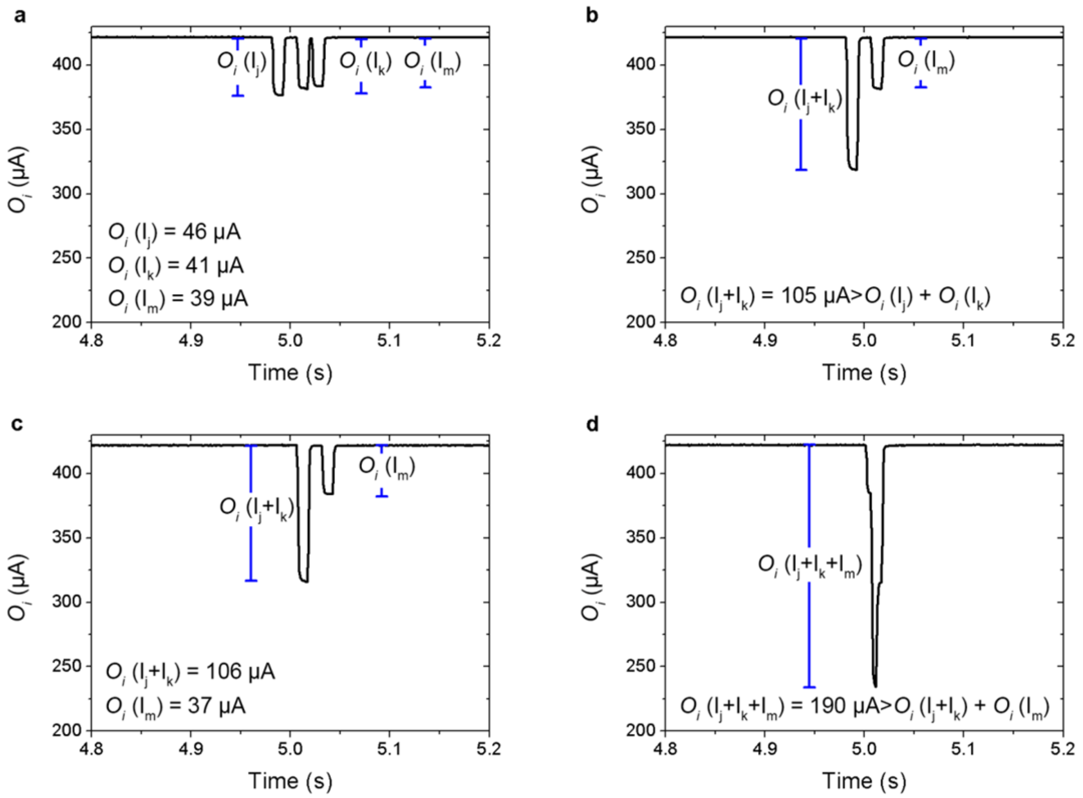

Supplementary Figure 7. Super-linear summation in coincidence detection measurements. (**a**)-(**b**) The sum two individual local inputs $O_i(I_i)$ and $O_i(I_k)$ (~ 87 μA) is smaller than the overall output $O_i(I_i + I_k)$ (~ 105 μA). (**c**)-(**d**) The sum of $O_i(I_j + I_k)$ and $O_i(I_m)$ (~ 143 μA) is smaller than the overall output $O_i(I_i + I_k + I_m)$ (~ 190 μA).

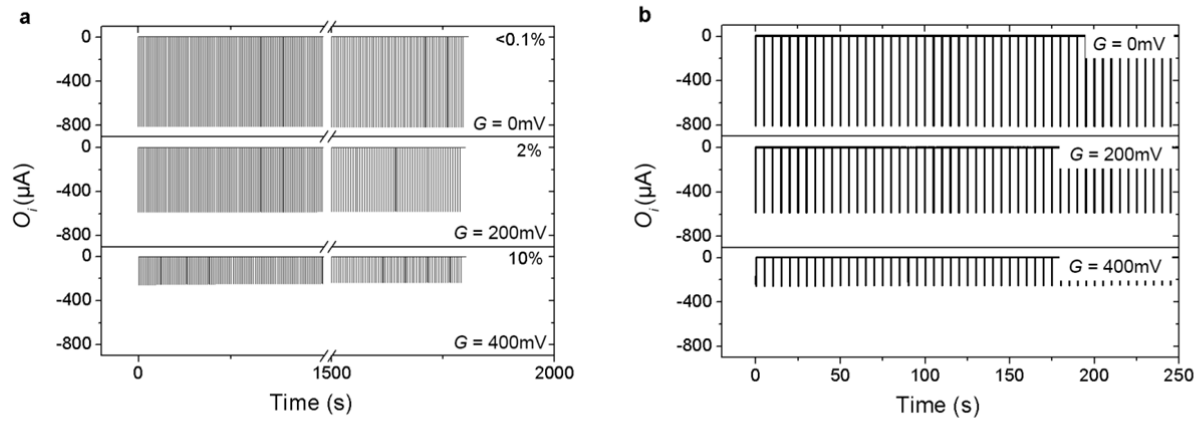

Supplementary Figure 8. Endurance measurements. Endurance measurements of the output O_i for applying a train of pulses (amplitude of 100 mV, width of 50 ms and period of 5 s) at the local input I_i (at coordinates (1, 1)) for global gate voltages $G = 0 – 400$ mV. (**a**) $N = 360$ cycles. (**b**) $N = 50$ cycles.

Supplementary Notes

Supplementary Note 1 - Gate voltage vs electrolyte concentration spatial mappings of local output O_i

Ionic concentration vs gate voltage spatial mappings are presented in **Supplementary Fig. 1**. A pulse is applied at every local input I_i (amplitude of 100 mV, width of 50 ms) and the amplitude of the corresponding output O_i is determined for different global DC gate voltages (G = 0 – 700 mV) and electrolyte concentrations (DI water – 100 mM NaCl). The global gating regulates more efficiently the I/O transmission for larger global voltages and with the presence of higher concentrations of ionic species. Thus the most effective route for the global regulation of every local I/O transmission is through the 'diagonal' of the diagram of **Supplementary Fig. 1**.

Supplementary Note 2 - Influence of the location of the gate on global gating

Regarding location of the gate in the device grid, two extreme regimes can be referred here. One extreme is when the gate electrode has much smaller dimensions than the PEDOT:PSS active area of a device and this regime was recently studied.[1] In the latter work, the output of the OECT was dependent on the distance between the gate and the drain electrode, and this dependency allowed the demonstration of orientation selectivity with a multi-gated OECT. The channel dimensions of the multi-gated device were in the range of ~ cm and the gates were approximately 4 times smaller. The opposite extreme is studied in the present work. Here, the channel width and length is $W \times L = 50 \times 50\ \mu m^2$, while the gate dimensions are much larger and in the range of ~ mm. In this case, the output of the device is practically independent on the distance between the global gate and the channel.

The difference between these two regimes can be understood quantitatively according to a previously published work by D. A. Bernards, G. G. Malliaras et., al.[2] According to this work, the relation between the electrolyte potential V_{EL} and the gate potential V_G is given by:

$$V_{EL} = \frac{V_G}{1 + {C_C}/{C_G}} \tag{1}$$

where C_C and C_G is the channel and the gate capacitance respectively. For large difference in the dimensions between the gate and the channel (i.e., the present case), $C_G >> C_C$ and thus $V_E \sim V_G$. This means that the whole gate voltage drops to the channel and the electrolyte resistance (or the gate-channel distance) is not a determinative parameter for the output of the device. For comparable dimensions (not applicable in this case) between the gate and the channel, capacitances C_C and C_G are also comparable and $V_E < V_G$. This results in a partial drop of the V_G at the electrolyte, and in this case gate-channel distance affects the output response of the device.[1]

Supplementary Fig. 2 shows the output O_i of the device at coordinates (1, 1) for the application of a pulse at the local input I_i (amplitude of 100 mV, width of 50 ms) and for various locations of the gate on the 4 × 4 grid (with dimensions of ~ 15x15 mm^2). The output O_i is practically independent on the exact location of the gate, at least within the dimensions of the grid.

Supplementary Note 3 - AC signals as global clocks

The I/O transmission of the grid of 4×4 devices for a global, AC signal is investigated in the **Supplementary Fig. 3**. Similarly to the single device measurements of **Fig. 3**, a train of pulses is applied at the I_i terminal of every device (amplitude of 100 mV, width of 50 ms, period of 5 s), a global AC signal is applied at G terminal (amplitude of ± 600 mV, frequency of 1 mHz) and the resulting spiking output response O_i of every device is measured as a function of time. The mapping of every local output amplitude O_i of the grid, at different moments of time of the global AC signal is depicted in **Supplementary Fig. 3**. The output response of the grid is regulated globally in a synchronized manner and the I/O transmission can be modulated with a periodicity between the extreme values 0 – O_{MAX}. Therefore, the AC signal is considered as synchronization function, or a global clock.

Supplementary Note 4 - Synchronization functions

The global oscillation of **Fig. 3(c)** is a result of the amplitude modulation (AM) of a 1 Hz signal with a 3 Hz signal. According to the formulation, the overall result of AM of a carrier signal (with frequency f_c) with a modulation signal (with frequency f_m) without phase difference φ (= 0) is given by:[3]

$$y(t) = ct_1 \cdot \sin(2\pi f_c t) + ct_2\left[\sin\left(2\pi(f_c + f_m)t\right) + \sin\left(2\pi(f_c - f_m)t\right)\right] \quad \text{(S2)}$$

Where ct_i is a constant and t is time. For f_c = 1 Hz and f_m = 3 Hz, it comes out the resulting AM modulation exhibits the harmonics of f_c = 1 Hz, $f_c + f_m$ = 4 Hz and $|f_c - f_m|$ = 2 Hz. In fact, these are the basic harmonics that are observed in **Fig. 3(c)**.

In **Fig. 3 (c)**, a global oscillation with a positive offset (in the range 500 mV to 900 mV) is chosen, in order to operate the device in the sub-threshold regime (i.e., G > 600 mV, or the 'OFF' state, see also **Fig. 1(d)**) during the majority of the global oscillation, and only during the basic harmonic of the AC signal (i.e., 1 Hz) the device is operated close to the threshold regime (G ~ 600 mV). This definition of the global oscillation is the key feature for synchronization of an input train of pulses at the local input I_i, with the basic harmonic of the global oscillation at 1 Hz, close the threshold regime. The output current O_i can dramatically be increased by using a steeper global oscillation below the threshold voltage (see **Supplementary Fig. 4**).

Supplementary Note 5 - OECT characteristic curves

Supplementary Fig. 5(a) shows typical, single device, $I_{DS} - V_{DS}$ measurements in typical transistor configuration (3-terminal configuration). The $I_{DS} - V_{DS}$ characteristic of the OECT1 is modulated by using a V_{GS} voltage (using a AgCl gate electrode), which exhibits a typical OECT behavior.[4] Regarding the present work, the I/O suppression with a global gate voltage, can be understood quantitatively by taking into account the typical $I_{DS} - V_{DS}$ of **Supplementary Fig. 5(a)**.

Moreover, another device (i.e., OECT2) can be used for gating the OECT1. **Supplementary Fig. 5(b)** shows similar $I_{\mathrm{DS}} - V_{\mathrm{DS}}$ measurements (for OECT1) for different 'gate' voltages at the OECT2, V_{OECT2} (applied at the source terminal of OECT2, while the drain is floating). **Supplementary Fig. 5(b)** shows that every OECT in the grid (in this case OECT2) effectively acts as a gate electrode for OECT1 through the electrolyte. Although this 'secondary' gating is less effective when compared with the AgCl electrode case (the $I_{\mathrm{DS}} - V_{\mathrm{DS}}$ is partially suppressed), it provides a quantitative explanation of the soft connectivity between individual devices through the electrolyte continuum.

Supplementary Note 6 - Measurement procedure of coincidence detection

The measurement procedure of the construction of the time correlation mapping of the output O_i, $t_k - t_j$ vs $t_m - t_j$ (refer also to **Fig. 5(b)**) is described thereafter. A voltage pulse ($V_{\mathrm{I}} = 0.5$ V, $t_{\mathrm{P}} = 10$ ms) is applied at three local inputs (I_j, I_k, I_m) with variable time intervals $t_k - t_j$ (for the $\mathrm{I_k}$ input) and $t_m - t_j$ (for the I_m input) in respect to a reference time t_j of the I_j input and the output amplitude O_i of the ith device (for $V_{\mathrm{O}} = 0.05$ V) is determined in every case (**Supplementary Fig. 6(a)**). The time correlation mapping of **Fig. 5(b)** was constructed following the procedure that is described below: the time interval of the pair of pulses at I_m and I_j terminals (= $t_m - t_j$) was kept constant and the time interval of the I_k terminal was varied, creating ($t_m - t_j$ = constant, $t_k - t_j$) arrays. Following the above procedure, for different $t_m - t_j$ = constant intervals, the surface of **Fig. 5(b)** is scanned and constructed (**Supplementary Fig. 6(b)-(f)**, O_i vs $t_k - t_j$ for different $t_m - t_j$ time intervals).

Supplementary Note 7 - Super-linear summation

An example of the raw data of the measurement of coincidence detection is presented in **Supplementary Fig. 7**, as resulted by applying pulses at the local inputs I_j, I_k and I_m for various time intervals and by defining the amplitude of the output current O_i. **Supplementary Fig. 7(a)** and **(b)**, show that the sum two

individual local inputs $O_i(\mathrm{I_i})$ and $O_i(\mathrm{I_k})$ is smaller than the overall output $O_i(\mathrm{I_i} + \mathrm{I_k})$, when inputs I_j and I_k are time-synchronized. Similarly, in **Supplementary Fig. 7(c)** and **(d)**, the sum of $O_i(\mathrm{I_j} + \mathrm{I_k})$ and $O_i(\mathrm{I_m})$ is also smaller than the overall output $O_i(\mathrm{I_i} + \mathrm{I_k} + \mathrm{I_m})$. Therefore, the overall output response O_i is slightly super-linear summation of the individual local inputs.

Supplementary Note 8 - Endurance measurements

Supplementary Fig. 8(a) shows the endurance measurements of the output O_i as a function of time for applying a train of pulses (N = 360 cycles, amplitude of 100 mV, width of 50 ms and period of 5 s) at the local input I_i (at coordinates (1, 1)) for global gate voltages G = 0 – 400 mV. **Supplementary Fig. 8(b)** shows the same measurement in more detail (N = 50 cycles). The output of the device is quite stable after 360 cycles. For G = 0 mV, the amplitude of O_i is quite stable (< 0.1% degradation), while for G = 400 mV the amplitude of O_i displays only a small degradation (~ 10 %).

Supplementary References

1 Gkoupidenis, P., Koutsouras, D. A., Lonjaret, T., Fairfield, J. A. & Malliaras, G. G. Orientation selectivity in a multi-gated organic electrochemical transistor. Sci. Rep. **6**, 27007, (2016).

2 Bernards, D. A. *et al.* Enzymatic sensing with organic electrochemical transistors. J. Mater. Chem. **18**, 116-120, (2008).

3 https://en.wikipedia.org/wiki/Amplitude_modulation.

4 Bernards, D. A. & Malliaras, G. G. Steady-State and Transient Behavior of Organic Electrochemical Transistors. Adv. Funct. Mater. **17**, 3538-3544, (2007).
